# Differential Expression of Wnt Pathway Genes in Sporadic Hepatocellular Carcinomas Infected With Hepatitis B Virus Identified With OligoGE Arrays

**DOI:** 10.5812/hepatmon.6192

**Published:** 2013-01-23

**Authors:** Xiaoyan Lin, Qiangxiu Wang, Zhixin Cao, Ming Geng, Yongcheng Cao, Xiaohong Liu

**Affiliations:** 1 Department of Pathology, Provincial Hospital Affiliated to Shandong University, Jinan, China; 2 Department of Pathology, General Hospital of Jinan Military Command, Jinan, China

**Keywords:** Carcinoma, Hepatocellular, Hepatitis B Virus, Oligonucleotide Array Sequence Analysis, Gene Expression Profiling, Disheveled Proteins

## Abstract

**Background:**

Epidemiological evidence has clearly indicated that chronic infection with the hepatitis B virus (HBV) is the major risk factor for developing hepatocellular carcinoma (HCC). Nonetheless, the mechanisms by which HBV contributes to the pathogenesis of HCC have not been fully elucidated.

**Objectives:**

Our aim was to characterize differential gene expression profiles related to the Wnt signaling pathway between primary tumor and adjacent normal tissues in HCC patients with concomitant HBVinfection .

**Materials and Methods:**

An oligoGEArray® (an oligonucleotide-based gene expression array platform) containing 126 Wnt signaling pathway-related genes was used to compare gene expressions between primary HCC and adjacent non-tumorous liver tissues from 10 patients with HCC. Selected differential genes were identified with real-time RT-PCR and immunohistochemistry (IHC). In particular, the protein of the differential gene DVL3 (disheveled, dsh homolog 3 [Drosophila]) was chosen to investigate whether it is up regulated in primary tumor correlated with the clinic pathological characteristics of HCC patients. For this purpose we examined 56 HCC tissue samples via IHC for the presence of DVL3 protein.

**Results:**

Sixteen genes were identified with significant differential expression between HCC and adjacent non-tumorous liver tissue. These genes have been previously associated with the Frizzled signaling pathway, cell cycle, transcription, or protein degradation. All (100%) of the tumor samples results from 56 HCC patients tested were positive for DVL3 via IHC. Based on the intensity of DVL3 immunoreactivity, 25 (44.6%) and 31 (55.4%) of the patients were classified aslow and high-DVL3, respectively, which correlated with tumor stage (P = 0.029).

**Conclusions:**

This study clarified a number of Wnt pathway-related genes which are dysregulated in HBV-associated HCC. These genes may be contributedto the frequent activation of the Wnt signaling pathway. Our results promote the role of the Wnt signaling pathway in HBV-associated HCC.

## 1. Background

Hepatocellular carcinoma (HCC) is the most common primary hepatic tumor and the third leading cause of cancer-related deaths in humans, with nearly 600,000 deaths annually worldwide (1, 2). Epidemiological evidence has clearly demonstrated that chronic infection with the hepatitis B virus (HBV) is the major risk factor for developing HCC (3), especially in southeastern Asia and Sub-Saharan Africa (3-5). Nonetheless, the mechanisms by which HBV contributes to the pathogenesis of HCC have not been fully elucidated. The Wnt signaling pathway is critical and highly conserved for proper embryonic development (6). However, aberrant activation of the classic Wnt pathway has been shown to play an important role in the development of many types of human cancers (6). A hallmark of Wnt pathway activation is the elevation of β-catenin in the cytoplasm and its further transport into the cell nucleus. Besides the canonical pathway, the Wnt signaling system also has alternative routes, generally known as the non-canonical pathway. These include Wnt-induced alterations in Ca2+ uptake and protein kinase C activation (7). Mutations in components of the Wnt signaling pathway, such as the genes adenomatous polyposis coli (APC), β-catenin (CTNNB1), and the axins (AXIN1 and AXIN2), promote activation of the non-canonical pathway and thereby contribute to the initiation and promotion of carcinogenesis. Regarding HCC, Kondo et al.(8) reported that 18% to34% of patient samples had exon (3) mutations in the CTNNB1 gene. In particular, the elevated expression of β-catenin was observed in 58.4% of HBV-associated HCCs and significantly correlated with large tumor size, poor histological grade, and high tumor grade, suggesting that the altered Wnt signaling pathway may play a crucial role in the pathogenesis of the HBV-associated HCC (9).

## 2. Objectives

The present study characterized the differential expression profiles of 126 genes related to the Wnt pathway between primary tumor and adjacent normal tissues in patients with HBV-associated HCC. Our results could broaden our understanding of the role of the Wnt signaling pathway in HBV-associated HCC.

## 3. Materials and Methods

### 3.1. Patients and Specimens

Between January 2009 and September 2010, 56 primary HCC specimens and surrounding non-neoplastic tissues (taken 5 cm away from the tumor edge) were collected from patients who had been undergone curative surgery at the General Hospital of Jinan Military Command. 51 were men and 5 women, with a median age of 51 years (range: 23-76 years). Of these, 37 cases had serum alpha-fetoprotein (AFP) ≥ 30 µg/L, and 50 had positive findings for hepatitis B surface antigen (HBsAg).

 None of these patients had positive findings of HCV infection or had undergone prior chemotherapy or radiotherapy. On gross examination, in 5 cases the tumor was < 2 cm, whereas in 51 cases was > 2 cm (mean, 5.1 cm; range, 1.0-13 cm). The clinic pathological findings were classified in accordance with the tumor-node-metastasis (TNM) staging system for malignant tumors of the World Health Organization and International Union against Cancer. All tumor tissues were diagnosed histopathologically by at least two trained pathologists. The resected tumors and matched non-cancerous tissue specimens used for microarray analysis and mRNA detection were immediately frozen in liquid nitrogen. The Ethics Committee of General Hospital of Jinan Military Command approved the study protocol. Informed written consent was obtained from each patient before the operation.

### 3.2. RNA Extraction and Processing for Microarray Analysis

Microarray analysis was performed, as described previously (10) using RNA isolated from tumor tissues and matched non-neoplastic tissues derived from 10 HCC patients, with some modifications. The clinical characteristics of these patients are listed in Table 1. In brief, total RNA was isolated from tissues using Trizol reagent (Invitrogen, Carlsbad, CA, USA) in accordance with the manufacturer’s suggested protocol (11) and then quantified with a spectrophotometer. Using the True-Labeling AMPTM Linear RNA amplification kit (Super Array Bioscience, Qiagen), the mRNA was reverse-transcribed into cDNA and converted into biotin-labeled cRNA using biotin-16-UTP via in vitro transcription. The purified cRNA probes were then hybridized to the pretreated OligoGEArray® Human Wnt Signaling Pathway arrays (Cat. No. OHS-043; Super Array Bioscience) containing 126 Wnt-related genes. After washing steps, array spots binding cRNA were detected using the chemiluminescence method (manufacturer’s protocol). The differentially expressed genes between HCC tissues and adjacent normal tissues were filtered based on fold-change cutoffs of ≤ 0.5 and ≥ 2 for down regulated and up regulated genes, respectively.

**Table 1:** Clinical Features of the Ten Patients With HBV-Associated HCC

Patient No.	Gender	Ages	TNM stage	HBsAg	Cirrhosis
**1**	Male	68	T4	Positive	Yes
**2**	Male	37	T4	Positive	Yes
**3**	Male	41	T3	Positive	Yes
**4**	Male	57	T3	Positive	Yes
**5**	Male	32	T3	Positive	Yes
**6**	Female	63	T2	Positive	Yes
**7**	Male	73	T2	Positive	Yes
**8**	Male	58	T3	Positive	Yes
**9**	Male	57	T3	Positive	Yes
**10**	Male	49	T2	Positive	Yes

Abbreviations: HCC, hepatocellular carcinoma; HBV, hepatitis B virus; HBsAg, hepatitis B surface antigen.

### 3.3. Quantitative Real-Time RT-PCR

Quantitative real-time RT-PCR (qRT-PCR) was performed as described previously (12). Reverse transcription was performed on 1 μg of total RNA from each sample using the MMLV Reverse Transcriptase 1st-Strand cDNA Synthesis Kit (Illumina), by following the manufacturer’s instructions. qRT-PCR was performed using SYBR Green in a Rotor-gene 3000 thermal cycler (Corbett Research, Sydney, Australia).

 The PCR primer sequences were designed for the selected genes and β-actin based on the gene sequences reported in GenBank and were chemically synthesized (Table 2). The specificity of the PCR was confirmed by examining the dissociation reaction plot subsequent to qRT-PCR. β-actin served as the constitutive control. PCR of each sample was conducted in triplicate. Data was analyzed using the comparative cycle threshold (Ct) method.

**Table 2:** Primer Sequences Usedfor qRT-PCR

Primer	Sequences	Annealing Temperature, °C	Product Size ,bp
**FZD2-F**	5’-CATCGTCATCGCTTGCTACT-3’	58	252
**FZD2-R**	5’-CTGTTGGTGAGGCGAGTGTA-3’		
**DVL3-F**	5’-AGGTGCCTATGCAAGTTCA-3’	58	138
**DVL3-R**	5’-TGTGCGAGGTTTAAGGTCTA-3’		
**PYGO2-F**	5’-GCTGCTAACGATGGGTGAC-3’	58	116
**PYGO2-R**	5’-AAGCCAGTGGAAACAAGGAC-3’		
***β-actin-F***	5’-CCTGTACGCCAACACAGTGC-3’	58	211
***β-actin-R***	5’-ATACTCCTGCTTGCTGATCC-3’		

Abbreviations: FZD2, Frizzled homolog 2; DVL3, disheveled 3; PYGO2, Pygopus homolog 2.

### 3.4. Immunohistochemistry (IHC)

Fifty-six HCC tissues were fixed in formalin, embedded in paraffin, and cut into 4 μm-thick sections. Sections were pretreated, dewaxed in xylene, and hydrated before antigen retrieval. After endogenous peroxidase inhibition, sections were incubated with a polyclonal antibody against human DVL3 (1:100; ab76081, Abcam, Hong Kong) overnight at 4 °C. After thorough washing with phosphate-buffered saline (PBS), corresponding secondary antibodies were applied and incubated at room temperature for 30 min (13). Reaction products were visualized by incubation with 3,3’-diaminobenzidine (DAB) and then counterstained with hematoxylin. Negative controls were achieved by substituting the primary antibody with isotype-matched irrelevant antibody.

### 3.5. Evaluation of Immunostaining

DVL3 staining was judged by two pathologists (Geng M and Cao YC) who were blinded to theclinical details related to the patients. Immunostaining was expressed as the percentage of stained cells to the total number of cells, and assigned to one of the four categories: 0, 0%; 1, 0% to 10%; 2, 10% to 50%; and 3, > 50%. The intensity of immunostaining was graded on a semi-quantitative scale (0-3): 0, negative; 1, weakly positive; 2, moderately positive; and 3, strongly positive. The two scores were multiplied and the product was defined as the IHC score. Final IHC scores below 4 indicated a low-level DVL3 expression; while more than 5was considered as high-level.

### 3.6. Statistical Analysis

Pearson's correlation analysis was performed to compare microarray and qRT-PCR data. The chi-squared test was used to analyze the relationship between DVL3 expression and clinic pathological characteristics. All statistical analyses were performed with SPSS version 11.0 software (SPSS, Chicago, IL, USA). A P value below 0.05 was considered statistically significant.

## 4. Results

### 4.1. Differential Wnt Pathway Gene Expression Between HCC and Adjacent Normal Tissues

We used OligoGEArray® Human Wnt Signaling Pathway arrays comprises 126 probe sets designed to measure human Wnt-related mRNAs to compare the gene expression profiles of primary HCC tumor and matched non-neoplastic tissues. We identified sixteen genes (12.7%, 16/126) which differed in expression significantly in at least six paired tissues. Of these, seven genes were up regulated (Table 3) and nine genes were down regulated (Table 4) in HCC tissues compared to the adjacent normal specimens. Remarkably, frizzled homolog 2 (FZD2) was overexpressed in nine of the HCC tissues, and pygopus homolog 2 (PYGO2) and split-hand/split-foot malformation 3 (SHFM3) were both downregulated in nine HCC tissues compared to their matched normal tissues. These differentially expressed genes are specifically related to the Frizzled signaling pathway, cell cycle, transcription, or protein degradation.

**Table 3:** Seven UpRegulated Genes (> 2-Fold Increase) in HCC Tissues Compared to the Matched Normal Tissues

Gene	Genbank ID	Symbol	Description
**Frizzled 2**	NM_001466	FZD2	G-protein coupled receptor which binds to Wnt proteins
**Casein kinase I isoform delta**	NM_001893	CSNK1D	Serine/threonine protein kinases
**Disheveled, dsh homolog 3**	NM_004423	DVL3	Protect β-catenin from *phosphorylated by kinase*
***Ras***** homolog gene family, member U**	NM_021205	RHOU	*Ras* homolog gene family, member U
**Secreted frizzled-related protein 4 **	NM_003014	SFRP4	A member of the SFRP family which acts as soluble modulators of Wnt signaling.
**WNT1-inducible signaling pathway protein-1 **	NM_003882	WISP1	Involved in cell growth
**Wingless-type MMTV Integration site family, member 3**	NM_030753	WNT3	A member of the Wnt *gene* family of secreted proteins

Abbreviation: HCC, hepatocellular carcinoma.

**Table 4:** Information for the Nine Down-Regulated Genes (< 2-Fold Increase) in HCC Tissues Compared to Their Matched Tissues

Gene	Genbank ID	Symbol	Description
**Pygopus 2**	NM_138300	PYGO2	Transcription factor
**Split-hand/split-foot malformation 3**	NM_022039	SHFM3	Involved in the ubiquitin pathway
**Adenomatous polyposis coil 2**	NM_005883	APC2	Tumor suppressor
**Fos-like antigen 1**	NM_005438	FOSL1	Transcription factor
**Naked cuticle 1 homolog (*Drosophila*)**	NM_033119	NKD1	A component of Wnt signaling pathway, unknown function
**Naked cuticle 2 homolog (*Drosophila*)**	NM_033120	NKD2	A component of Wnt signaling pathway, unknown function
**Protein phosphatase 2, catalytic subunit, alpha isozyme**	NM_002715	PPP2CA	Involved in cell cycle and apoptosis
**Transducin-like enhancer of split 4**	NM_007005	TLE4	A component of Wnt signaling pathway
**Wingless-Type MMTV Integration Site Family, Member 10A**	NM_025216	WNT10A	A member of Wnt signaling pathway

Abbreviation: HCC, hepatocellular carcinoma.

### 4.2. Validation of the Data From the Microarray by qRT-PCR

To validate the differentially expressed genes identified through expression microarray analysis, we performed qRT-PCR for FZD2, PYGO2, and DVL3, which had been found differentially expressed in at least six HCC tissues. There was a strong association between the two methods for all three of the genes which were compared, as revealed by the P values and Pearson’s correlation coefficients (FZD2, 0.987; DVL3, 0.920; PYGO2, 0.947; P< 0.001 for all). Compared to the adjacent normal tissues, the mRNA expressions of DVL3 and FZD2 were up regulated, whereas PYGO2 mRNA was down regulated in the HCC tissues (All P< 0.05).

### 4.3. DVL3 Protein Expression in HCC

**Figure 1:**
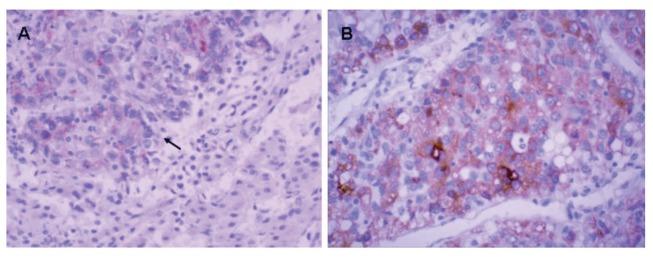
IHC Detection of DVL3 Protein Expression in HCC Tissue Samples Positive Findings of DVL3 Immunostaining Was Mainly Localized in the Cytoplasm of HCC Cells. A) DVL3 Immunostaining Is Moderately Positive in HCC (Arrow) and Negative Expression in Adjacent Non-Neoplastic Tissue; B) DVL3 Immunostaining Is Strongly Positive in HCC Tissue. Original Magnification: 200× in A; 400× in B.

To determine whether the up regulation of DVL3 protein is linked to the clinical variables of HCC patients, we examined the expression of DVL3 protein in 56 HCC tissue samples with IHC. Positive staining which indicated the presence of DVL3 protein was mainly localized to the cytoplasm of tumor cells (Figure 1). Using DVL3 immunoreactive intensity as the determinant, 25 (44.6%) patients were classified as low-DVL3 and 31 (55.4%) as high-DVL3. In addition, there was a significant positive correlation between the levels of DVL3 mRNA and DVL3 protein in the primary tumor tissues and the matched adjacent normal tissues (P = 0.031). Table 5 summarizes the association between DVL3 expression and the clinic pathological variables of the HCC patients. The expression of DVL3 protein was significantly correlated with the patients` tumor stage (P = 0.025). However, statistical analyses failed to reveal any significant correlations between DVL3 expression and other clinic pathological factors.

**Table 5:** The Relationship Between DVL3 Expression and Clinic pathological Features in Hepatocellular Carcinomas

	No.	DVL3 Immunoreactivity		*P *value
		High, No.	Low, No.	
**Gender**				NS
Male	51	28	23	
Female	5	3	2	
**Age, y**				NS
< 51	21	12	9	
≥ 51	35	19	16	
**Serum AFP level, µg/L**				NS
< 30	19	11	8	
≥ 30	37	20	17	
**HBsAg**				NS
Positive	50	27	23	
Negative	6	4	2	
**Tumor size**				NS
≤ 2 cm	5	3	2	
> 2 cm	51	28	23	
**Histological grade**				NS
Well differentiated	8	5	3	
Moderately differentiated	30	18	12	
Poorly differentiated	18	8	10	
**Liver cirrhosis**				NS
Absent	16	9	7	
Present	40	22	18	
**T classification**				0.025
T1	2	0	2	
T2	27	11	16	
T3	15	10	5	
T4	12	10	2	
Total	56	31	25	

Abbreviation: NS, not significant.

## 5. Discussion

The Wnt signaling pathway is essential to a variety of biological processes. Dysregulation of this pathway is a common hallmark of many human cancers, including HCC (14, 15). The data reported here clearly demonstrate that a group of genes directly or indirectly related to the Wnt pathway are dysregulated in HCC. The expression of FZD2 was up regulated in HCC tissues compared to the matched non-neoplastic tissues. FZD2, a member of Frizzled receptor family, contains a seven-transmembrane domain that serves as binding sites for many Wnt proteins (16). Casein kinase 1Δ (CSNK1D) is a member of the casein kinase I family (CKI), and CKI phosphorylates APC, axin, and β-catenin. CKI phosphorylates β-catenin at serine 45, and thereby allows progressive phosphorylation with glycogen synthase kinase 3β (GSK3B) and ubiquitination(17). We detected upregulation of DVL3 in HCC tissues. DVL3 inhibits the activity of GSK3B directly and protects β-catenin from degradation, therefore contributes to abnormal cell proliferation (18). WISP1 (WNT1-inducible-signaling pathway protein 1), a downstream regulator in the Wnt pathway, and the well-known oncogenes RHOU and WNT3 were also up regulated in HCC tissues. Previous evidence clearly showed that the expression of SFRP4 (secreted frizzled-related protein 4) was up regulated in colorectal cancer tissues, but down regulated in other cancer types (19); we found it to be upregulated in HCC tissues. This suggests that SFRP4 has a cell-specific function in different tumors. We also found nine genes which are down regulated in HCC tissues compared to matched normal tissues. Of these, NKD1 (naked cuticle 1), NKD2 and TLE4 (transducin-like enhancer protein) participate in the Wnt signaling pathway, through mechanisms which are not completely understood. APC2, a well-known tumor suppressor gene, is down regulated in many tumors (20). We consistently observed that APC2 was also down regulated in our examined HCC tissues. The present study also indicated that the SHFM3 gene, which is involved in the ubiquitin pathway, was down regulated in HCC tissues, suggesting aberrant activation of the classic Wnt pathway. However, we found down regulation of PYGO2, which was founded to be up regulated in ovarian cancer (21). Moreover, FOSL1 (fos-related antigen 1), a downstream regulator in the Wnt pathway, was overexpressed significantly in HCC tissues. Understanding the exact roles of these genes in HCC requires further elucidation. In the present study, we performed qRT-PCR analysis of FZD2, PYGO2, and DVL3 to validate the microarray data. Moreover, we assessed the DVL3 protein expression in 56 HCC tissues with IHC. High-level of DVL3 expression was observed in 55.4% (31 of 56 cases) and correlated with tumor stage (P = 0.029). Collectively, our findings suggest that the DVL3 is up regulated in HCC tissues and positively participates in HCC progression. Nonetheless, further studies are needed to elucidate the molecular mechanisms by which the DVL3 participates in the development and progression of HCC, and to address whether the DVL3 could be used as a target for novel therapeutic approaches. In summary, the present microarray study revealed that a number of Wnt pathway genes are dysregulated in HBV-associated HCC, which may be contributed to the frequent activation of the Wnt signaling pathway. Our results may reveal further understanding of the role of the Wnt signaling pathway in HBV-associated HCC.

## References

[1] Aravalli RN, Steer CJ, Cressman EN (2008). Molecular mechanisms of hepatocellular carcinoma.. Hepatology..

[2] Llovet JM, Burroughs A, Bruix J (2003). Hepatocellular carcinoma.. Lancet..

[3] Mas VR, Maluf DG, Archer KJ, Yanek K, Kong X, Kulik L (2009). Genes involved in viral carcinogenesis and tumor initiation in hepatitis C virus-induced hepatocellular carcinoma.. Mol Med..

[4] Di Bisceglie AM (2009). Hepatitis B and hepatocellular carcinoma.. Hepatology..

[5] El-Serag HB, Rudolph KL (2007). Hepatocellular carcinoma: epidemiology and molecular carcinogenesis.. Gastroenterology..

[6] Logan CY, Nusse R (2004). The Wnt signaling pathway in development and disease.. Annu Rev Cell Dev Biol..

[7] Montcouquiol M, Crenshaw EB, 3rd, Kelley MW (2006). Noncanonical Wnt signaling and neural polarity.. Annu Rev Neurosci..

[8] Kondo Y, Kanai Y, Sakamoto M, Genda T, Mizokami M, Ueda R (1999). Beta-catenin accumulation and mutation of exon 3 of the beta-catenin gene in hepatocellular carcinoma.. Jpn J Cancer Res..

[9] Joo M, Lee HK, Kang YK (2003). Expression of beta-catenin in hepatocellular carcinoma in relation to tumor cell proliferation and cyclin D1 expression.. J Korean Med Sci..

[10] Salajegheh M, Kong SW, Pinkus JL, Walsh RJ, Liao A, Nazareno R (2010). Interferon-stimulated gene 15 (ISG15) conjugates proteins in dermatomyositis muscle with perifascicular atrophy.. Ann Neurol..

[11] Wang P, Xu TY, Guan YF, Su DF, Fan GR, Miao CY (2009). Perivascular adipose tissue-derived visfatin is a vascular smooth muscle cell growth factor: role of nicotinamide mononucleotide.. Cardiovasc Res..

[12] Wang P, Xu TY, Guan YF, Tian WW, Viollet B, Rui YC (2011). Nicotinamide phosphoribosyltransferase protects against ischemic stroke through SIRT1-dependent adenosine monophosphate-activated kinase pathway.. Ann Neurol..

[13] Wang P, Yang FJ, Du H, Guan YF, Xu TY, Xu XW (2011). Involvement of leptin receptor long isoform (LepRb)-STAT3 signaling pathway in brain fat mass- and obesity-associated (FTO) downregulation during energy restriction.. Mol Med..

[14] Moon RT, Kohn AD, De Ferrari GV, Kaykas A (2004). WNT and beta-catenin signalling: diseases and therapies.. Nat Rev Genet..

[15] Rattis FM, Voermans C, Reya T (2004). Wnt signaling in the stem cell niche.. Curr Opin Hematol..

[16] Katoh M (2005). WNT/PCP signaling pathway and human cancer (review).. Oncol Rep..

[17] Gao ZH, Seeling JM, Hill V, Yochum A, Virshup DM (2002). Casein kinase I phosphorylates and destabilizes the beta-catenin degradation complex.. Proc Natl Acad Sci U S A..

[18] Lee AY, He B, You L, Dadfarmay S, Xu Z, Mazieres J (2004). Expression of the secreted frizzled-related protein gene family is downregulated in human mesothelioma.. Oncogene..

[19] Lustig B, Behrens J (2003). The Wnt signaling pathway and its role in tumor development.. J Cancer Res Clin Oncol..

[20] Ma L, Wang HY (2006). Suppression of cyclic GMP-dependent protein kinase is essential to the Wnt/cGMP/Ca2+ pathway.. J Biol Chem..

[21] Wang HY, Liu T, Malbon CC (2006). Structure-function analysis of Frizzleds.. Cell Signal..

